# Could a plant derived protein potentiate the anticancer effects of a stem cell in brain cancer?

**DOI:** 10.18632/oncotarget.25090

**Published:** 2018-04-20

**Authors:** Camila Ramalho Bonturi, Helena Motaln, Mariana Cristina Cabral Silva, Bruno Ramos Salu, Marlon Vilela de Brito, Luciana de Andrade Luz Cost, Heron Fernandes Vieira Torquato, Natalia Neto dos Santos Nunes, Edgar Julian Paredes-Gamero, Tamara Lah Turnšek, Maria Luiza Vilela Oliva

**Affiliations:** ^1^ Biochemistry Department, Federal University of São Paulo, 04044-020, São Paulo - SP, Brazil; ^2^ Genetic Toxicology and Cancer Biology Department, National Institute of Biology, 1000, Ljubljana, Slovenia; ^3^ Biophysics Department, Federal University of São Paulo, 04039-032, São Paulo-SP, Brazil

**Keywords:** brain cancer, glioma, stem cells, inhibitors, invasion

## Abstract

Glioblastoma is the most aggressive brain tumor with poor overall survival bellow 2 years. The natural compounds with anti-cancer properties, are thus gaining attention for possible adjuvant GBM treatment. In various cancer models *Enterolobium contortisiliquum* Trypsin Inhibitor (EcTI) proved to have anti-cancer effects. Here, we investigated the EcTI effects on GBM U87 cells and on mesenchymal stem cells (MSC) compared to their direct coculture (MSC/U87). MSC are present in tumor stroma, modulating GBM cells phenotype, and also represent potential drug delivery vehicle due to their tumor tropism. We showed that in p53-wild type U87 cells, metabolic activity was less affected by EcTI as in MSC monocuture, but the metabolic rate of mixed coculture was significantly reduced at lower EcTI concentration. Under coculture condition, EcTI potentiated MSC induced cell cycle arrest, possible due to highly increased p53, p21 and lower D1 expression, but there was no effect on apoptosis. Accordingly, in the coculture EcTI also enhanced Ca^2+^ signalling mediated *via* bradykinin receptor 2, being associated with nitric oxide release that highly impaired proliferation and invasion. The mechanism did not seem to involve changes in cell adhesion but rather it down-regulated the β_1_ integrin signaling with associated p-FAK in U87 cells, both supporting inhibition of invasion. Finally, some cytokines were down-regulated, indicating that EcTI inhibition of signalling might be mediated by cytokines. In conclusion, these results indicate that in cocultured MSC/U87 cells EcTI impairs the metabolic activity, proliferation, and reduced invasion, possibly associated with observed cytokines secretion. In this context, we confirmed that the plant derived protein potentiated the anticancer effects, induced by MSC, as represented by GBM U87 cell line.

## INTRODUCTION

Glioblastoma (GBM) is the most aggressive among all brain tumors and represents 51% of glioma. Life expectancy upon diagnosis is generally less than 2 years due to its recurrence even after restrictive surgery, chemo and radiotherapy [1]. The treatment of GBM remains one of the biggest challenges in oncology. Advanced cell therapies employing normal human neural cells [2] and mesenchymal stem cells (MSC) [3] seem to be promising in preclinical experimental settings. MSCs are being studied as a tool for cancer treatment due to their tropism to tumors and immunomodulatory ability [4–6]. They secrete cytokines, growth factors, proteases, and several binding ligands that can alter the morphology, proliferative, and migratory behaviour of cancer cells [7, 8]. MSCs are also part of the glioma microenvironment, where together with other types of stromal cells, such as fibroblasts, immune cells, and endothelial cells [9] affect tumor progression. We previously observed in direct coculture that numerous gap junctions are formed between GBM cells and MSCs, followed by the over-expression of connexin 43 [10].

In the progression of GBM, proteases and their inhibitors participate not only in the degradation of extracellular matrix (ECM) [11] but also in the regulation of other processes by proteolytic trimming and activation or inactivation of several signalling proteins [12, 13]. Protease inhibitors have been widely used in the treatment of various pathologies including cancer [14]. The modulation of intercellular signalling pathways by members of the Kunitz protease inhibitors family has already been demonstrated suggesting that these should be considered as therapeutic agents [15–17]. The plant Kunitz-type inhibitor EcTI, isolated from *Enterolobium contortisiliquum* seeds (*Leguminosae* family, *Mimosidae* subfamily), has been previously studied in tumor models [18, 19]. Low doses of EcTI were shown to inhibit adhesion, migration, and invasion of gastric cancer cells through a decreased expression of active integrin β_1_ that lead to decreased FAK and Src phosphorylation and prevented the invadopodia formation [18]. Therefore, here we analyzed the effects of EcTI on MSCs and GBM cells cultures and their coculture. Our goal was to explore, if the potential anticancer effects of EcTI on U87 cells could be enhanced by MSCs in their direct coculture that would suggest a potential use of EcTI as an adjuvant agent in MSC-based therapy of glioma or other types of cancers in future.

## RESULTS

### EcTI inhibited metabolic activity and cell cycle

**Figure 1:**
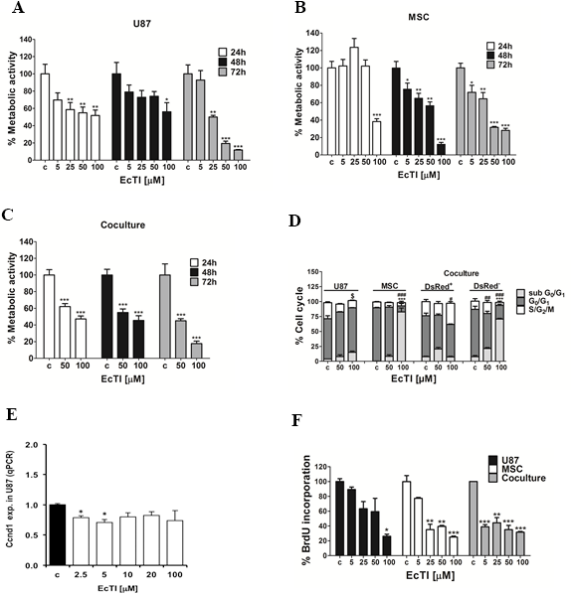
Effects of EcTI on cell metabolic activity and proliferation EcTI affects the viability of U87 cells in GBM **(A)**; MSCs **(B)**; and coculture **(C)**. Cells were treated with increasing concentrations of EcTI (5, 25, 50, and 100 μM) for 24 and 48 h and their viability was determined by metabolic rate using the MTT assay. Absorbance values were measured at 540 nm and normalized to the control of non-treated cells (c). Cell cycle percentage after EcTI treatment (50 and 100 μM) for 24 h **(D)**. Statistical analysis represents the relation between control (c) and EcTI concentrations, considering the symbol (^*^) for statistical within sub G_0_/G_1_, (^#^) G_0_/G_1_, (^$^) S/G_2_/M. **(E)** mRNA levels of CcDN1 in U87 cells treated with EcTI (2.5, 5, 10, 20, and 100 μM) for 72 h and presented relative to control non-treated U87 cells; **(F)** Effects of EcTI on the proliferation of MSC and U87 cells, and coculture with increasing EcTI concentrations. The percentage was measured by the ratio between treated and control cells. Error bars represent SD. ^*^p < 0.05, ^**^p< 0.005, ^***^p< 0.0005 were considered significant in the statistical analysis performed by the Tukey's test and one-way ANOVA.

**Figure 2:**
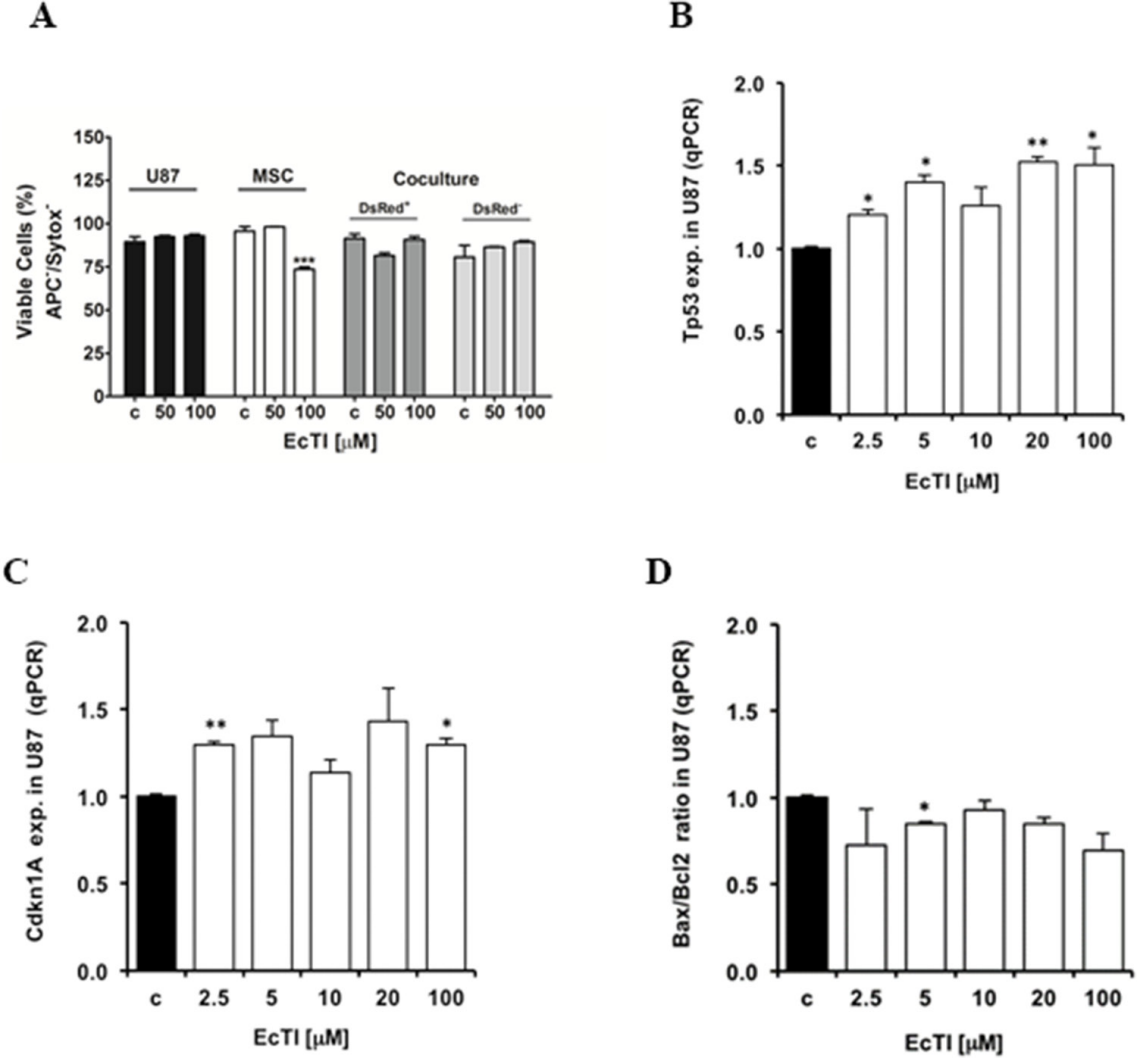
EcTI did not interfere in apoptosis Effect of EcTI on cell death **(A)** in GBM U87 cells, MSCs, and coculture after 24 h of treatment using 50 μM and 100 μM EcTI final concentrations. The cell death was performed as described in Material and Methods. Effect of EcTI analysis on the tumor protein p53, *Tp53* **(B)**; p21, *Cdkn1A* **(C)**; and *Bax/Bcl2* ratio **(D)**. mRNA levels in U87 cells treated with EcTI (2.5, 5, 10, 20, and 100 μM) for 72 h and presented relative to control non-treated U87 cells. The error bars represent SD (^*^p < 0.05, ^**^p< 0.005, ^***^p< 0.0005).

EcTI at 100 μM concentration impaired the metabolic activity of U87 cells in a time-dose-dependent manner, reaching nearly 50% of inhibition after 24 h, yet the metabolic activity was almost completely restored after 48 h, but after 72 h, the metabolic activity was drastically reduced by 90% (Figure 1A). Likewise MSC cells, although initially more resistant to EcTI than U87 cells, exhibited 50% reduction in metabolic activity at 50 μM EcTI at 48 h and 70% after 72h (Figure 1B). In direct coculture, 100 μM EcTI reduced the metabolic activity by 50% at 24 h, which remained reduced for 48 h and up to 72 h (reducing more than 80%) (Figure 1C). This data also denote that EcTI, being a protein was resistance to extracellular proteolysis by secreted proteases that has been demonstrated in U87/MSC crosstalk [29, 30]. Next, the effect of EcTI on the cell cycle, being one of the key hallmarks of cancer [31], accompanied by metabolic activity and proliferation rate of cancer cells. Cell cycle analysis of the U87 cells after EcTI treatment revealed about 10% increase in G_0_/G_1_ phase, and about 15% decrease of cells in S/G_2_/M phase. On the other hand, in the direct coculture, where labelled U87 cells (DsRed) [29], were used, an increase in S, G_2_ and M phase was observed, pointing on distinct effects of the inhibitor in mono and coculture conditions. MSC showed an increase in sub G_0_/G_1_ either in mono or direct coculture with U87(DsRed^−^) cells (Figure 1D). This is consistent with the observed decreased expression of *Ccnd1* gene (Cyclin D1) in U87 cells exposed to EcTI (Figure 1E). Cyclin D1 in the deregulated state is known to cause cell cycle arrest, as its intracellular translocation promotes G_1_/S transition [32]. Our data imply on U87 cell cycle arrest as the result of a decrease in cyclin D1 expression. The latter is most likely resulting from observed increase in cell cycle inhibitor *Cdkn1A* (p21) and *P53* gene expressions (Figures 2B, 2C), as described below.

### EcTI decreased cell proliferation

Cell proliferation was evaluated by BrDU incorporation after treatment with increasing EcTI concentrations. U87 cells showed decreased proliferation only at 100 μM EcTI, whereas the proliferation of MSCs was affected at all doses higher than 5 μM. These results are consistent with EcTI impairment of cellular metabolic activity described above. However, although the anti-proliferative EcTI effect in U87 cells was observed only at 100 μM cells, the proliferation decreased by nearly 60% at all EcTI concentrations in MSC/U87 cell coculture (Figure 1F).

### EcTI had no effects on apoptosis

EcTI exhibited no effect on apoptosis (indicated as negative Annexin-V and Sytox staining) in either U87 cells or in their coculture after 24 h of treatment (Figure 2A). However, a 30% decrease was observed in the viability of MSCs, but only at the highest EcTI concentration. This decrease was restored when MSCs were grown in direct coculture with U87 cells. The above is in line with the gene expression analyses in EcTI treated U87 cells, where elevated expression of *Tp53* (Figure 2B) and *Cdkn1A* (p21) (Figure 2C) gene were detected. U87 cells express the wild-type p53 tumor suppressor, which is known to induce DNA repair mechanisms, causes cell cycle arrest, and leads to apoptosis when damage repair is unsuccessful [31]. It seems likely that the up-regulation of p53 in U87 cells induced by EcTI activated the cell cycle inhibitor p21 expression with simultaneous cyclin D1 (Figure 1E). the latter downregulated and cyclin-dependent kinases 4/6 activity, which is crucial for RB protein phosphorylation and regulation of the G_1_/S phase transition [31, 33]. Consistent with EcTI having no effect on apoptosis induction in U87 cells (Figure 2A), only a slight decrease of the pro-apoptotic Bax/Bcl2 ratio was observed in U87 cells after 5 μM EcTI treatment, but there were no changes at even 100 μM EcTI concentration (Figure 2D).

Taken together, EcTI inhibited the metabolic activity of U87 and MSC cells in mono and direct coculture, slightly impaired their cell cycle, however, it did not induce cell death.

### EcTI effects on cell adhesion

**Figure 3:**
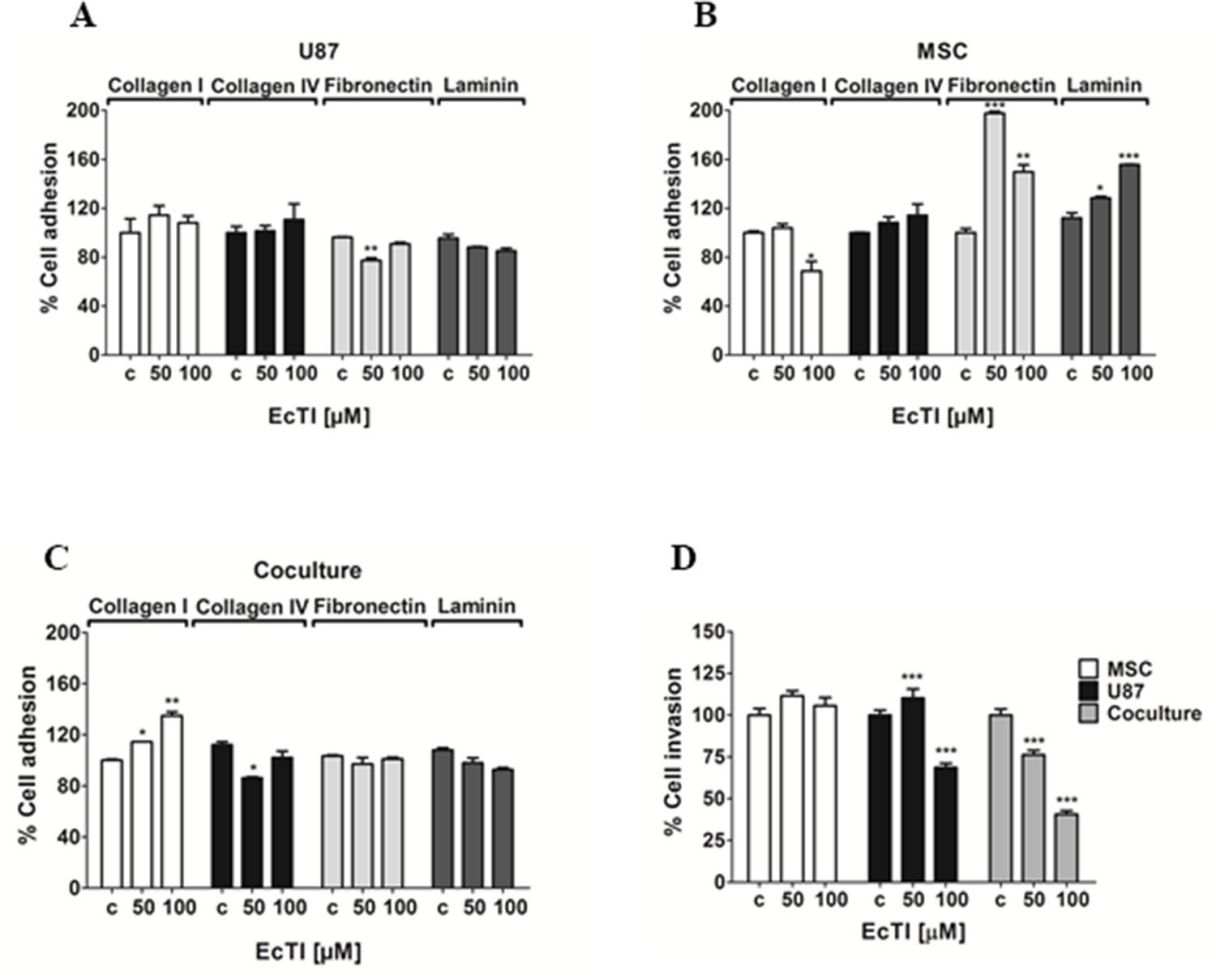
Effects of EcTI on cell adhesion and invasion EcTI in 50 and 100 μM differentially affects the adhesion of U87 **(A)**; MSC **(B)**; and coculture **(C)** coated with collagen I, collagen IV, laminin, and fibronectin. The absorbance values measured at 540 nm were normalized to control non-treated cells (c). EcTI effects on the invasion of mono-cultured GBM U87 cells, MSCs, and cocultured **(D)** were measured in Boyden chambers. Error bars represent SD (^*^p < 0.05, ^**^p< 0.005, ^***^p< 0.0005).

Adhesion to the extracellular matrix (ECM) is an essential step in tumor invasion process [34]. Therefore, we analyzed the effect of EcTI on the adhesion of MSC and U87 cells and their cocultures to the ECM proteins. The adhesion of U87 cells (Figure 3A) to all tested substrates, except fibronectin, remained unchanged upon EcTI treatment. Conversely, (Figure 3B) about 30% inhibition of MSC adhesion to collagen I was observed at the 100 μM EcTI treatment, whereas their adhesion to fibronectin and laminin highly increased. In direct coculture (Figure 3C), EcTI increased cell adhesion up to 35% only to collagen I. Direct MSC/I87 cell-to-cell contact in coculture may change the expression of various plasma membrane ECM binding proteins, including different types of integrins, membrane-anchored cadherins, and growth factor receptors. Out of these, we only observed an affect integrin β_1_, where EcTI impaired its expression in U87 cells and increased its level in MSC cells (see below).

### EcTI diminished cell invasion

Cell invasion is also an important hallmark of cancer and highly supporting the aggressiveness of GBM [36, 37]. EcTI decreased invasion of U87 cells by about 30% at 100 μM only. In contrast, EcTI highly impaired invasion of cocultured cells in a dose-dependent manner down to 50% at highest 100 μM EcTI treatment (Figure 3D). Noteworthy, the invasion of MSCs was not affected by EcTI suggesting that, in direct coculture, these cells may act in a synergistic manner with EcTI towards the decreased invasiveness of U87 cells by either paracrine, exosomal, or direct cell-cell molecular exchange [9, 10].

### EcTI effects on integrin β_1_ signalling

**Figure 4:**
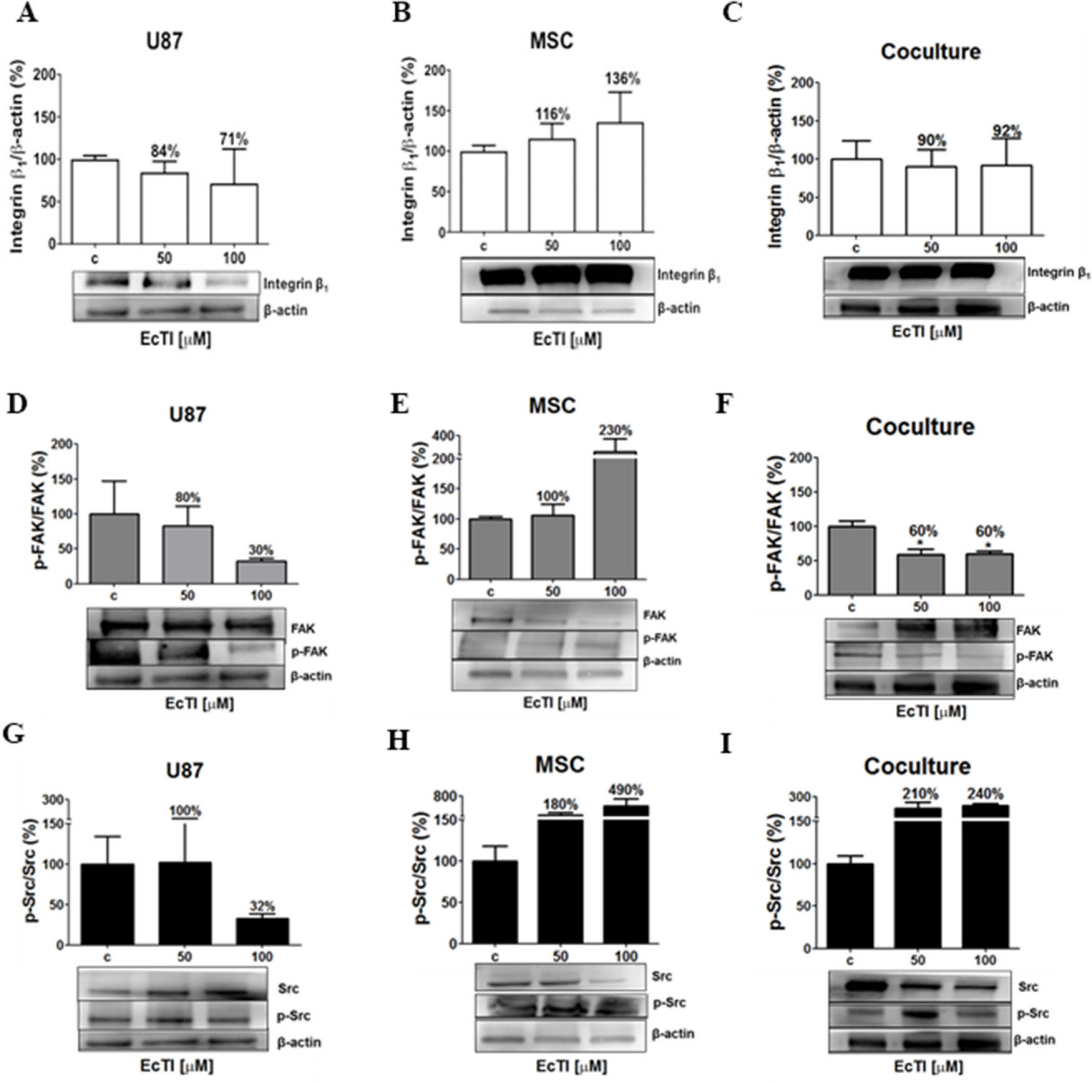
Effects of EcTI on integrin expression and intracellular signalling Integrin β_1_ protein expression: EcTI affects integrin β_1_ protein expression in GBM U87 cells **(A)**, MSCs **(B)**, and coculture **(C)**. The cells were treated with EcTI (50 and 100 μM) for 24 h. After SDS-PAGE separation of cell lysates and electrotransfer to PVDF membrane, the binding with anti-integrin β_1_ and anti-β-actin (as loading control) was performed and detected by chemiluminescence and relative to the integrin β_1_ expression quantitation that was performed by densitometry analysis in the Image J software with β-actin normalization. The percentage of protein expression in treated cells is presented relative to its expression in non-treated control (c) cells. Kinase signalling: The expressions of the ratio p-FAK/FAK in U87 **(D)**; MSC **(E)**; and coculture **(F)**; p-Src/Src in U87 **(G)**; MSC **(H)**; and coculture **(I)** were analyzed, as described in Material and Methods. All the proteins were normalized by β-actin. Error bars represent SD (^*^p < 0.05, ^**^p< 0.005, ^***^p< 0.0005).

Besides mediating adhesion and invasion, integrins are responsible for mediating apoptosis (anoikis), survival, proliferation, and invasion [38]. At 100 μM concentration, EcTI decreased the expression of the β_1_ integrin protein in U87 cells by 29% (Figure 4A) and increased it in MSCs by 36% (Figure 4B). In monocultures, a decrease in U87 cells may be correlated with their reduced adhesion to fibronectin. Similarly, increased adhesion to fibronectin and laminin observed in MSCs after EcTI treatment correlated with increased β_1_ integrin signalling. No change in β_1_ integrin expression was observed in the coculture possibly due to compensating the opposite effects in each cell type the overall expression (Figure 4C). Taken together, down-regulation of β_1_ integrin content in U87 cells was associated with an inhibition of their invasion. Although the β_1_integrin expression remained unchanged in the coculture, a distinguished decrease in invasion was noted in cocultured cells upon the EcTI treatment. In MSC alone, despite increased β_1_ integrin expression in MSCs, no change in their invasion was noted. These findings corroborate the mechanism of EcTI activity, proposed by de Paula et al. [18] involving EcTI binding to the β_1_ integrin subunit and thereby inhibiting the invasion of tumor cells.

### EcTI effects on the FAK/Src signalling pathway

FAK phosphorylation attracts the Src–SH protein docking. This complex subsequently activates phosphorylation cascades that may trigger various cellular responses [39, 40]. Therefore, we investigated the phosphorylation of FAK and Src kinases (p-FAK and p-Src, respectively). The EcTI treatment at 100 μM concentration reduced the levels of p-FAK/FAK ratio by 70% in U87 cells and by 40% in the coculture (Figure 4D and 4F) what is consistent with its anti-proliferative effects. It is also in line with EcTI inhibition of invasion in cocultured cells. In contrast, 100 μM EcTI doubled the p-FAK/FAK ratio in MSCs (Figure 4E), corroborating the observation that EcTI did not affect MSCs invasion (compare with Figure 3D). Conversely, the ratio of p-Src/Src was found highly increased in MSCs and coculture exposed to 100 μM EcTI (Figure 4H and 4I), whereas it was found decreased in U87 cells (Figure 4G). This implies on p-Src/Src involvement in EcTI inhibition of U87 cells invasion. In summary, EcTI diminished β_1_ integrin activation of p-FAK/FAK and p-Src/Src kinases in U87 cells alone, where their invasion was probably impaired due to lower FAK kinase activation of p-Src [40]. These results suggest that EcTI impairs the invasion of cocultured cells by inhibiting p-FAK/FAK signalling.

### EcTI effects on proteases expression

**Figure 5:**
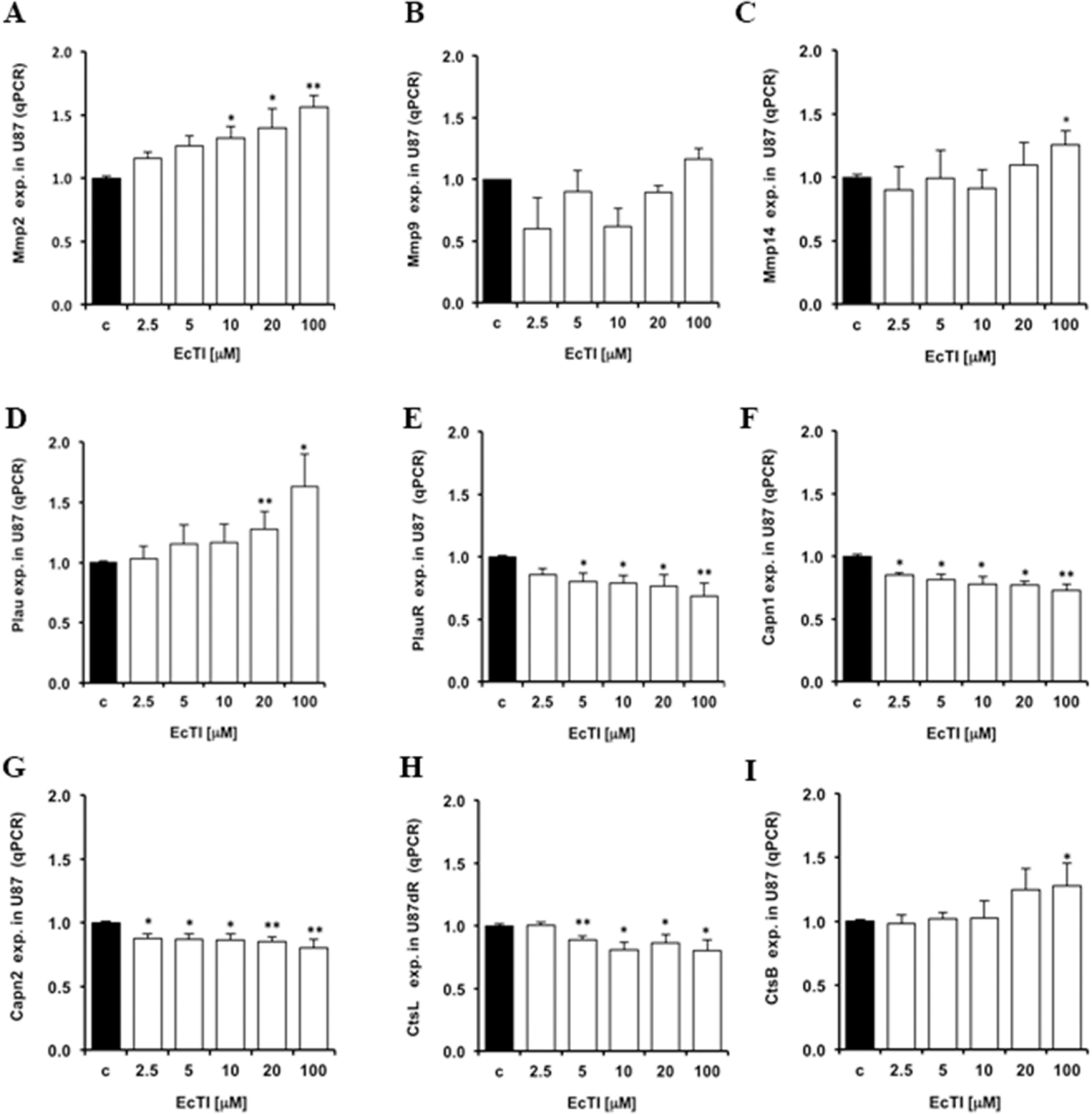
Changes in protease gene expression caused by EcTI treatment The EcTI effects on the protease expression in U87 cells were measured by qRT-PCR analysis and compared to non-treated control (c) cells.The effect of EcTI on the expression of the metalloproteases *Mmp2* **(A)**, *Mmp9* **(B)**, *Mmp14* **(C)**, urokinase plasminogen activator, *Plau* **(D)** and its receptor, *PlauR* **(E)**, Calpain 1, *Capn1* **(F)**, Calpain 2, *Capn2* **(G)**, Cathepsin L, *CtsL* **(H)**, and Cathepsin B, *CtsB* **(I)**. *Error bars* indicate SD. ^*^p < 0.05, ^**^p< 0.005, ^***^p< 0.0005 were considered significant.

Tumor cells’ invasion is associated with altered expression, localization, and activities of proteases that by cleaving various proteins also play a role in other processes denoted as hallmarks of cancer [41]. Thus, exogenous inhibitors of natural origin represent a valuable tool to normalize increased protease activities when these are causative of cancer progression [17]. With respect to invasion, we investigated a set of metallo-serine- and cysteine proteases, reported to be part of invasion-related proteolytic cascade. The matrix metalloprotease *Mmp2* gene expression was increased, in a dose-dependent manner, upon EcTI treatment in U87 cells (Figure 5A), whereas the *Mmp9* gene expression was not affected (Figure 5B). However, the plasma membrane associated MMP-14 (MT1-MMP), known to proteolytically activate both MMP-2 and MMP-9, was also found up-regulated (Figure 5C) in U87 cells upon the EcTI treatment. Thus, we may conclude that EcTI possibly enhanced MMP-2 activity resulted from extracellular signalling that increased the *Mmp2* gene expression in EcTI treated U87 cells and was correlated with MMP-14 activity. The expression of the plasminogen activator urokinase gene (*PLAU*) and its uPAR receptor (*PLAUR)* are relevant for the invasion cascade, due to their role as activators of several MMPs [42, 43]. The binding of urokinase to anchored-uPAR plasma membrane allows for extracellular plasminogen conversion into plasmin, which may activate MMPs. By the same token, uPAR is responsible for the indirect induction of pericellular proteolysis via binding with some integrins (α_V_β_5_ and α_V_β_1_) [43]. Here, EcTI up-regulated the urokinase gene expression (Figure 5D), however, it down-regulated its *PlauR* receptor expression (Figure 5E) in U87 cells, suggesting the prevention of required binding and activation of urokinase. On the other hand, EcTI down-regulated a set of proteases, such as calpains (Capn) which are calcium-dependent cysteine proteases that play a role in cancer call migration [44]. Capn 1 and 2 show different activation responses to μM and mM Ca^2+^ concentrations, respectively. Capn 2 has also been reported to be involved in the invasion and migration of GBM cells [44]. We demonstrated that EcTI decreased the *Capn1* (Figure 5F) and *Capn2* (Figure 5G) gene expression in U87 cells. Likewise, the gene expression of invasion-related cysteine cathepsins [11, 13], cathepsin L (*CtsL*) was found decreased (Figure 5H), whereasknown invasion promoting cathepsin B (*CtsB*) [29] was found slightly increased in U87 cells upon EcTI treatment (Figure 5I). Taken together, EcTI upregulated metallo proteases, urokinase and cathepsin B, but down regulated urokinase receptor (uPAR), calpains, and cathepsin L genes in U87 cells. Yet, in EcTI treated U87 cells, these proteases may not be active and/or may not be relevant for invasion process in the cocultures [29, 42] , since despite their decreased expression the invasion of U87 cells and the coculture, was found highly impaired.

### EcTI downregulated bradykinin receptors expression in coculture

**Figure 6:**
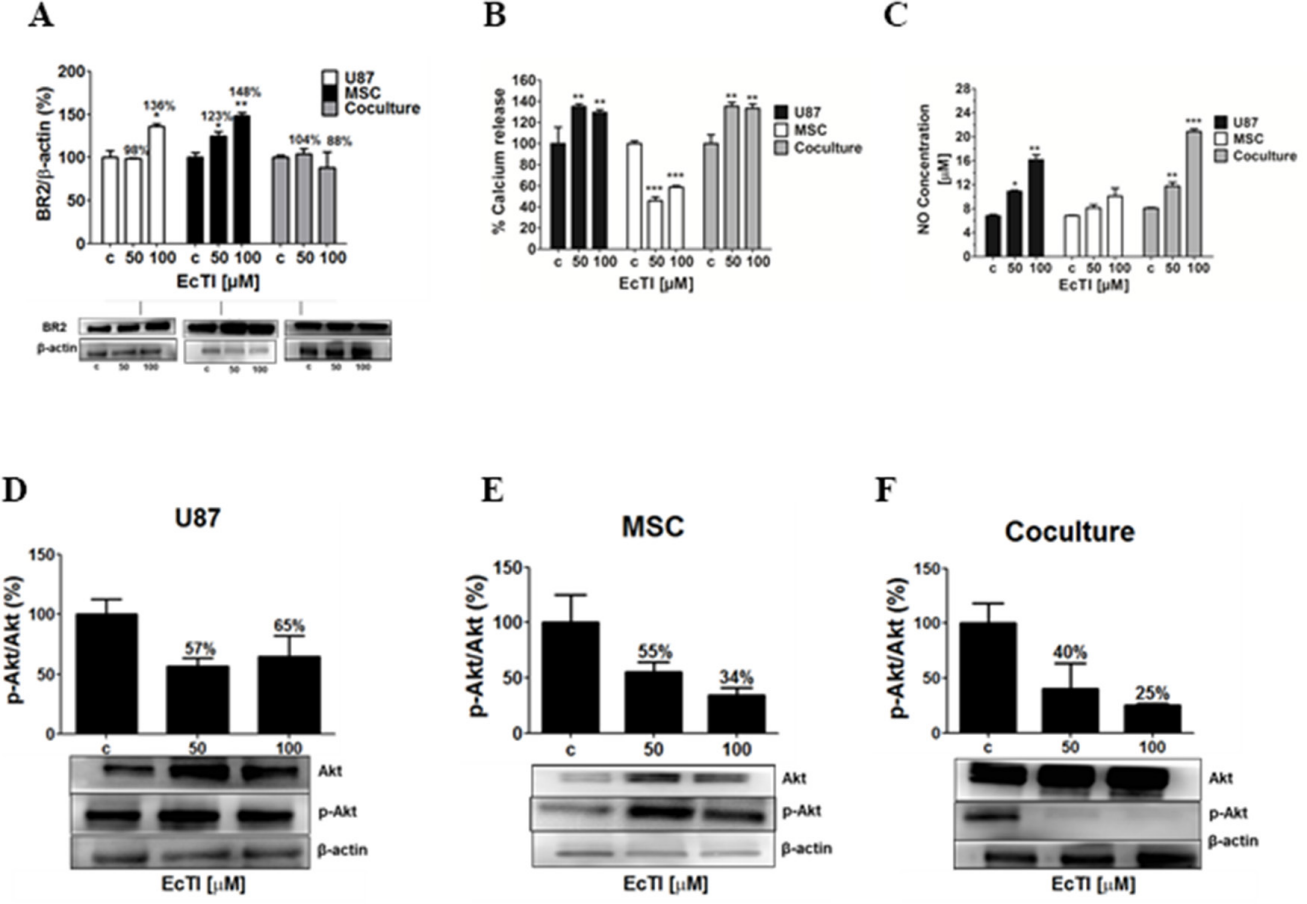
The Effect of EcTI on the expression of the bradykinin receptor 2 (BR2), oxide nitric (NO), and Akt protein Expression of BR2 in the cell culture of cancer U87 cells, mesenchymal stem cells (MSC), and coculture **(A)**. The quantification of the relative BR2 expression in each sample was performed by densitometry analysis in the Image J software with β-actin normalization. The effects of EcTI on calcium release **(B)** and detection of NO concentration **(C)** in U87 cells, MSCs, and coculture. Expression of the ratio p-Akt/Akt in MSC **(D)**, U87 **(E)**, and coculture **(F)** compared to non-treated control (c) cells. The error bars represent SD. ^*^p < 0.05, ^**^p< 0.005, ^***^p< 0.0005 were considered significant.

A significant down-regulation of the expression of urokinase receptor PlauR, needed for urokinase activity, may have affected other transmembrane proteins *via* fluidic transmembrane diffusion. We have recently [21] elucidated that U87 cells via direct crosstalk affect invasion of MSC, which is possibly activated by the kallikrein-kinin system and mediated *via* bradykinin receptors (BRs) [42, 43, 45]. Therefore, we measured the BR2 expression in U87 cells, MSC and in the coculture. EcTI enhanced the level of BR2 in U87 cells and in MSC (Figure 6A). Contrary, EcTI lowered the BR2 levels by 12% at 100 μM in MSC/U87 cell coculture. This suggests possible BR2 internalization and/or its degradation by secreted MMPs. Considering these results, EcTI downregulated the BR2 levels in the coculture, which then correlated with the inhibition of invasion observed in these cocultures (compare with Figure 3D).

### EcTI effects on calcium, nitric oxide release, and the Akt signalling pathway

Calcium (Ca^2+^) is an important second messenger that influences cell migration, metastases, cell-to-cell communication, and apoptosis [46]. The cytosolic Ca^2+^ levels were measured in treated MSC and U87 cells and in their cocultures to evaluate the impact of EcTI on calcium mobilization. EcTI increased Ca^2+^ fluxes in U87 cells and coculture by nearly 40% (Figure 6B). Oppositely, EcTI decreased Ca^2+^ fluxes by about 50% in MSCs (Figure 6B). Enhanced Ca^2+^ mobilization may result in nitric oxide (NO) production/release because elevated Ca^2+^ levels are required for the nitric oxide synthase (NOs) activity. Here, we demonstrated a dose-dependent increase in NO release, which doubled in U87 cells and nearly tripled in coculture at 100 μM EcTI, however, no such effect was observed in MSCs (Figure 6C). These data parallel the Ca^2+^ release. Altogether, these results suggest that the inhibition of the metabolic activity in U87 mono and cocultured cells (Figure 1) after treatment with EcTI, may have resulted from the cytosolic Ca^2+^ mobilization and increased production of nitric oxide (NO).

Akt is the cytosolic kinase that is active in tumor cell survival, apoptosis, and invasion. Akt is also involved in the regulation of Ca^2+^ flux and eNOS activity [50–52]. Akt was not activated by EcTI treatment, as the p-Akt/Akt ratio decreased in EcTI treated U87 cells, MSC and in the coculture (Figure 6D, 6E and 6F). The decreased ratio of p-Akt/Akt in treated MSCs correlated with their Ca^2+^ down-regulation. Likewise, the decreased expression of BR2 in cell coculture may have accounted for 75% reduction in p-Akt/Akt ratio and may be related to the inhibited invasion and proliferation of these cells after EcTI treatment.

### EcTI affects cytokines release

**Figure 7:**
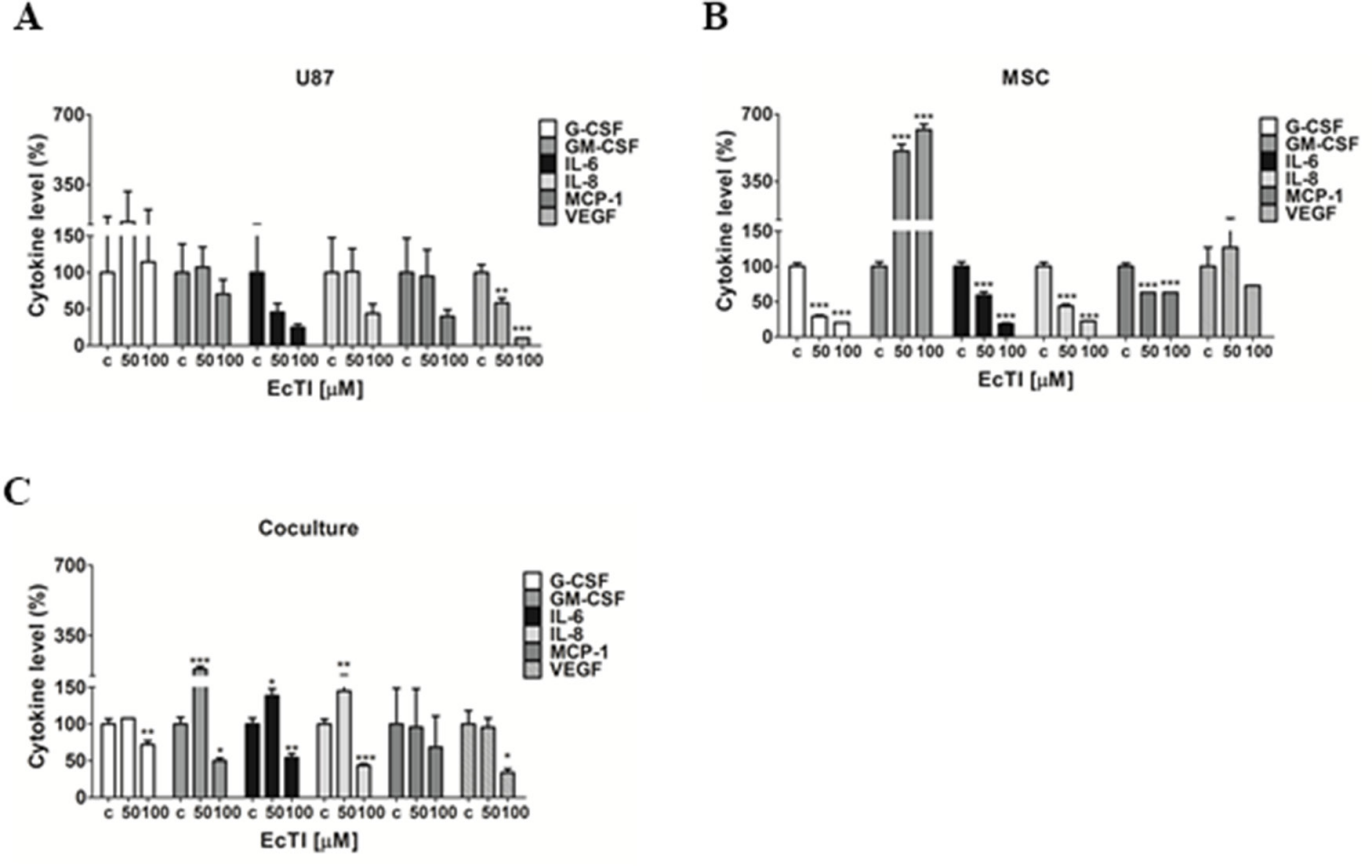
EcTI affects cytokine secretion EcTI affects the secretion of the following cytokines G-CSF, GM-CSF, IL-6, IL-8, MCP-1, and VEGF from GBM U87 **(A)** and MSC **(B)** cultures, and their coculture **(C)** treated with EcTI (50 and 100 μM) for 24 h. Cytokine profiling was performed using treated media (50 and 100 μM) compared to non-treated control (c) cells. The error bars represent SD (^*^p < 0.05, ^**^p< 0.005, ^***^p< 0.0005).

Several pro-inflammatory cytokines play a role in cancer cell motility, apoptosis, and angiogenesis, and are elevated in patients with GBM [53]. Among them, cytokines IL-6 and IL-8 activities are related to glioma progression [54]. GM-CSF was shown to stimulate proliferation and growth of GBM [54], whereas VEGF induces angiogenesis. Here we determined the level of a set of cytokines in the culture medium derived from U87 cells (Figure 7A), MSC (Figure 7B), and coculture (Figure 7C), all treated with EcTI for 24 h.

EcTI significantly decreased VEGF secretion at the 100 μM concentration in U87 cells (Figure 7A), though slightly inhibited the secretion of almost all cytokines in treated U87 cells, except G-CSF. EcTI decreased the secretion of G-CSF, IL-6, IL-8, and MCP-1 in MSC, however, it significantly increased the secretion of GM-CSF. Intriguingly, we observed increased levels of GM-CSF, IL-6, and IL-8 at 50 μM EcTI treatment in coculture, whereas these, together with VEGF were decreased upon 100 μM EcTI treatment. Altogether, our data suggest that EcTI inhibition of metabolic activity and invasion in the direct cocultures may also be associated with the altered levels of cytokines expression and secretion.

## DISCUSSION

Kunitz-type plant protease inhibitors are known to display anticancer effects triggered by different biological and molecular mechanisms. The plant Kunitz-type inhibitor EcTI, isolated from *Enterolobium contortisiliquum* seeds has been shown previously to affect the adhesion, migration, and cell invasion of gastric cancer cells [18]. Therefore, we investigated EcTI effects on GBM cells, as their high rate of local invasion *in vivo* represents the key obstacle to successful treatment, and as yet the effects of this inhibitor on any malignant astrocytic cell line have not been studied. Here, we demonstrated that EcTI also inhibited invasiveness of U87 cells, taken as model GBM cell line, and reduced the activity of the integrin β_1_ subunit and impaired FAK, Src as well and Akt signalling. In their study on gastric carcinoma cells, de Paula et al. [18] have demonstrated that EcTI decreased the expression and disrupt the cellular organization of molecules involved in the maturation of invadopodia, such as integrins, cortactin and neuronal Wiskott-Aldrich syndrome protein, simultaneously with integrin-dependent cell adhesion signalling kinases. The adhesion on various matrix proteins of U87 cell line was not significantly altered, whereas EcTI have presented an impressive effect upon adhesion of gastric cancer cells. Interestingly, in U87 monoculture EcTI at lower concentrations actually enhanced invasion, and increased the levels of metalloproteases, MMP2, MMP9 and MMP14, as well as cathepsin B, whereas cathepsin L and calpains were slightly but significantly downregulated. This clearly proves that EcTI did not act as protease inhibitor of invasion-related proteases, and at higher levels impaired invasion *via* integrin downregulation. In contrast, in gastric carcinoma lines, the decrease of MT1-MMP (MMP14) was observed, explained as being the consequence of a decrease in functional activity of invadopodia. The discrepancy observed in adhesion and protease regulation upon EcTI treatment between gastric carcinoma and U87 may be explained by their epithelial vs a more mesenchymal histological origin, respectively. Further, in U87 cells’ viability decreased with increasing EcTI concentration, and the proliferation diminished due to the arrest in G_1_/S phase, associated with slight decrease in Cyclin D1, but a more significant increase in cell cycle inhibitor p21 gene expression. Noteworthy, U87 express wild type p53 protein, which is most likely the master regulator of these events. However, there was no sign on apoptosis associated with EcTI treatment.

The key novelty of the presented research, however, is that we focused on the effects of EcTI on tumor interactive microenvironment. This is associated with so called inter-tumor heterogeneity, due to the presence of various types of infiltrative normal stromal cells, fibroblasts, immune cells and endothelial cells, as well as mesenchymal stem cells (MSC) [4, 6, 10]. Here we demonstrated that in direct MSC coculture with U87 (MSC/U87) EcTI efficiently inhibited their metabolic activity and proliferation. The treatment of the coculture with EcTI reduced the cell invasion, p-FAK/FAK and p-Akt/Akt signalling in paralel to altered proteases and bradykinin receptor expression, as discussed below. This data corroborated recent report by Breznik et al. [29] on 3D spheroids between MSC and each the two GBM cell lines U87 and U373, revealing that MSC inhibited invasion of U87, but not of U373. The present results clearly demonstrate that the MSC inhibition of invasion of U87 was potentiated by addition of EcTI. In addition, Breznik et al. [29] also found that in MSC/U87 coculture, the proteases, such as MMP9 and MMP14 were downregulated, but no change in the levels of cathepsin B, calpain 2 and MMP2 was observed. Pillat et al. [21] reported that indirect and direct effects of MSC on U87 cells were associated with increased expression levels of both, kininogen receptors B1 (BR1) and BR2. It was hypothesized that kinin receptor expression was induced in MSC/U87 co-cultures, enhancing calcium influx. Montana and Sontheimer [42] showed that bradykinin via B2R-induced [Ca^2+^]_I_ transients enhanced invasion of GBM cells the formation of small bleb-like protrusions at the plasma membrane, which stimulated an amoeboid phenotype of cell migration. In contrast to the above, we found here that EcTI down-regulated BR2 levels in coculture, which correlated with their inhibited invasion. Together with Ca^2+^ release results, this suggests that EcTI mediated inhibition of metabolic activity and decreased DNA synthesis in U87 cells in coculture may have resulted from cytosolic calcium mobilization and increased NO production. NO is a permeable gas associated with diverse cellular events, including tumor suppression. It can have a therapeutic effect, when used as a treatment prior to chemotherapy to sensitize GBM cells [47]. Furthermore, elevated NO concentrations have an anti-proliferative effect and may even induce apoptosis and increase the permeability of the blood-brain barrier [47]. *In vivo*, the NO production by healthy adjacent tissues inhibited tumor invasion [48], regulated blood flow, and reduced tumor volume [49]. Taken together, based on our results, a mechanism of EcTI biological effects on MSC/U87 cocultures may involve the BR2, Ca^2+^ influx and NO production.

EcTI therefore affects the MSC re-programming of the GBM cell. Moreover, MSC may even fuse with U87 cells, when directly interacting with them to form coculture hybrids enabling direct molecular exchange and even genetic reprogramming [10, 35, 56]. Indeed, Mercapide et al. [9] demonstrated heterotypic fusions to occur between MSC and U87 cells when in direct contact, resulting in enhanced proliferation of tumor hybrids. This corroborates our recent data, where Oliveira et al. [56] demonstrated that even up to 30% of U87 fuse with MSC, the latter often get degraded within U87 by a process called entosis or cannibalism [9], whereas interestingly, only 3% of U373 cells fuse with MSC [35].

The inhibition of proliferation and invasion in U87 cells in co-cultures by EcTI was also associated with a decreased secretion of pro-inflammatory cytokines, both secreted by U87 and MSC cells, except G-SCF, which was increased. The secretion of CCL2/MCP-1 have been previously shown increased in indirect and direct coculture [6] and was responsible for the inhibition of U87 cell invasion [55]. Here, no additional effects of EcTI in CCL2 were observed, being possibly sufficient to mediate inhibitory effects on MSC/U87 coculture. These observations add to the complexity of the intracellular signalling affected by EcTI in U87 cells and their direct coculture with MSCs.

In conclusion, modern therapeutic strategies for cancer chemotherapy involve the administration of drug mixtures, enabling the administration of lower doses of each drug than if applied individually. Our study, although still at an experimental level, proves that at least the proneural/mesenchymal type of glioblastoma, as modelled here by U87 cells, in the heterogonous tumor i.e. in the presence of MSCs, would be effectively treated by EcTI to reduce its aggressive behaviour. Finally, to answer the title question: Yes, the results presented here are quite promising and highlight the potential of EcTI as an antitumor agent in future, possibly as an adjuvant in cell therapy by MSC as cells vectors. At present, this protein may contribute to better understanding of the pathological processes involved in glioblastoma and useful as a tool to investigate heterogeneous tumour interactions in response to this natural product.

## MATERIALS AND METHODS

### Materials

Penicillin, streptomycin, and geneticin (G418), fetal bovine serum (FBS), non-essential amino acids, and Na-pyruvate (0.1 M) were purchased from Gibco® (Gaithersburg, USA). The Dulbecco's Modified Eagle's Medium (DMEM) with 1 g/l glucose, L-glutamine, 3-(4,5-dimethylthiazol-2-yl)-2,5-diphenyltetrazolium bromide (MTT), dimethyl sulfoxide (DMSO), protease inhibitor cocktail (8×10^-5^ M aprotinin, 0.0014 M E-64, 0.104 M AEBSF, 0.004 M bestatin, 0.0015 M pepstatin A and 0.002 M leupeptin), phosphatase inhibitor cocktail (sodium molybdate, sodium tartrate, sodium orthovanadate and imidazole), and trypsin 0.25% (v/v) were obtained from Sigma (St. Louis, MO, USA). Rabbit antibodies against human integrin β_1_ (#4706, catalog number), Akt (#4691), p-Akt (#4060), Src (#2108), p-Src (#6943), FAK (#3285) and p-FAK (#3283), β-actin (#4967), and IgG conjugated with horseradish peroxidase (#7073) derived from rabbit or mouse were purchased from Cell Signalling Technology (Danvers, USA). The mouse monoclonal antibody against human B2 bradykinin receptor (BR2) was obtained from BD Biosciences (610451, California, USA). The micro BCA™ and Super signal® west pico chemiluminescent substrate, Annexin APC and Sytox green were purchased from Thermo Scientific (Rockford, Illinois, USA).

### EcTI purification

It was performed as reported by de Paula et al. [18]. Briefly, *Enterolobium contortisiliquum* seeds (1:40 w/v) were homogenized at 25°C in 0.05 M Tris/HCl buffer at pH 8.0. The homogenate was filtered, centrifuged at 4000 x g for 15 min at 4°C, and the supernatant heated at 60°C for 10 min. The supernatant was subsequently precipitated in cold acetone (80% v/v) at 4°C for 30 min without agitation. The solution was centrifuged at 4000 x g for 20 min at 4°C and the precipitate was solubilized in 0.05 M Tris/HCl buffer at pH 8.0 and applied to a DEAE-Sepharose column equilibrated with 0.1 M Tris/HCl buffer at pH 8.0. The adsorbed proteins were eluted with 0.1 M Tris/HCl buffer at pH 8.0 containing 0.15 M NaCl and subjected to affinity chromatography using a Trypsin-Sepharose column equilibrated in 0.1 M Tris/HCl buffer at pH 8.0. The adsorbed protein (EcTI) was eluted with 0.5 M KCl/HCl solution at pH 2.0 with immediate pH neutralization with 1.0 M Tris/HCl solution. The fractions (1 ml/min) were dialyzed in water, lyophilized, resuspended in water, filtered, and subjected to size exclusion chromatography using a Superdex 75 10/300 GL column (GE Healthcare). This column was equilibrated with 0.05 M Tris/HCl buffer at pH 8.0 containing 0.15 M NaCl at a flow rate of 0.5 ml/min using a ÄKTA Avant (GE Healthcare). The absorbance of the fraction (1 ml) was monitored at 280 nm. The homogeneity of inhibitor purification was assessed by reversed-phase chromatography in a C_18_ column using a linear gradient (0-100%) of 90% acetonitrile in 0.1% trifluoroacetic acid (TFA) for 60 min in a flow rate of 0.7 ml/min. The molecular mass of EcTI was assessed by 5–12% SDS-polyacrylamide gel electrophoresis and the inhibitory activity against trypsin (1 μg/10 μl) was checked by a colorimetric assay (A_405_) using the Bz-Arg-pNa (BAPA) substrate (1 μg/25 μl) [20]. EcTI was concentrated and dialyzed in milli-Q water for the cell experiments.

### Cell culture

Experiments were performed using the U87-MG human glioblastoma cell line (ATTC® HTB-14™) that was stably transfected with the pCMV DsRed-Express2 plasmid as previously described in double-labeling U87 and MSC cells experiments [10, 21]. For the sake of comparison with those results, and because the transformation did not cause any obvious phenotypic changes, herein the cell was designated as U87.

Bone marrow-derived mesenchymal stem cells (MSC) were obtained from Lonza BioScience Walkerswille Inc. (Walkersville, MD). MSCs were cultured at 37°C and 5% (v/v) CO_2_ in DMEM medium supplemented with 0.002 M L-glutamine, 0.001 M Na-pyruvate, 1x non-essential amino acids, 100 μg/ml streptomycin, 100 IU/ml penicillin, and 20% (v/v) heat-inactivated fetal bovine, whereas U87 cells were cultured in 10% (v/v) heat-inactivated FBS and 1 mg/ml geneticin. The medium was changed every three days. MSCs at passages 4 – 10 and U87s at passages 50 – 72 were used in the experiments; all experiments performed used 10% FBS in the medium.

### Direct coculture experiments

Cells were mixed in 1:2.5 ratio (MSC: U87) for direct coculture and seeded in different plates according to the experiment.

### MTT assay

The metabolic activity indicating the viability of U87 and MSCs cells grown as mono and direct coculture was determined by the 3-(4.5-dimethylthiazol-2-yl)-2.5-diphenyltetrazolium bromide assay [22]. Monocultures were set up with 2,000 MSCs and 5,000 U87 cells seeded in 100 μl of media/well, in triplicates, and incubated at 37°C and 5% (v/v) CO_2_ for 24 h, 48 h and 72h, respectively, in the presence of EcTI. Direct coculture used the same number of cells mixed and seeded in 100 μl of media per well; media was changed after 24 h and EcTI was added (5-100 μM/200 μl/well). The MTT reagent (5 mg/ml) was added after 24 h and 48 h (two treatments); formazan crystals were dissolved in 100 μl of DMSO after 2 h of incubation. Absorbance was measured at 540 nm using a microplate reader (Spectra max Plus 384, Molecular Devices, CA, USA).

### BrDU Incorporation

Cell proliferation was evaluated by new DNA synthesis with bromodeoxyuridine intercalation according to the Cell Proliferation Elisa BrDU colorimetric kit (Life Sciences Inc.). Cells were harvested in 100 μl of media/well in a 96-well plate containing 2 × 10^4^ MSC and 5 × 10^4^ U87; the same amount of cells was used in the coculture. EcTI was added to each treatment well (5 μM-100 μM); plates were incubated at 37°C and 5% (v/v) CO_2_ for 24 h. BrDU was diluted to 10 μM final concentration/well and incubated for 4 h at 37°C and 5% (v/v) CO_2_. The medium was removed and 200 μl of fixing and denaturation solution was added to all wells; plates were incubated for 30 min at room temperature. The BrDU luminescence was detected by an anti-BrDU-peroxidase conjugated antibody at 405 nm in a FlexStation Multi-Mode Microplate Reader (Molecular Devices).

### Cell adhesion assay

Cell adhesion was evaluated in 96-well plates coated with: i) 100 μl laminin (4 μg), ii) fibronectin (4 μg) diluted in 0.01 M disodium phosphate buffer (0.0017 M KH_2_PO_4_, 0.0027 M KCl, and 0.14 M NaCl at pH 7.4), iii) collagen type I (8 μg), and iv) collagen type IV (4 μg) diluted in 2% (v/v) acetic acid. The wells were incubated with 100 μl of 1% (w/v) BSA in PBS at pH 7.4 for 1 h at 37°C and rinsed three times with 200 μl PBS at pH 7.4 [23]. U87 cells (5 × 10^4^ cells) and MSC cells (2 × 10^4^ cells) were either incubated as monocultures or coculture for 15 min with EcTI (50 and 100 μM) in a final volume of 100 μl. Suspensions of EcTI treated cells were transferred into coated wells and left to incubate at 37°C and 5% (v/v) CO_2_ for 4 h. Non-adherent cells were removed through a triple wash with PBS (100 μl each time) and the remaining cells were fixed with 100% (v/v) methanol for 40 min, washed with PBS, and stained with 1% (w/v) toluidine blue (diluted in 1% (w/v) sodium tetraborate) for 30 min. These cells were subsequently washed three times with PBS, followed by the addition of 1% (w/v) SDS and incubation for 30 min at 37°C. Absorbance was measured at 540 nm using a microplate reader (Spectra max Plus 384, Molecular Devices, CA, USA).

### Matrigel invasion assay

Millicell^®^ culture plate inserts (Millipore, MA, USA) with a pore diameter of 8.0 μm were placed into 24-well plates and coated with 100 μl of Matrigel (1:6 (v/v) in 0.01 M PBS at pH 7.4) (BD, San Diego, USA), which was allowed to polymerize for 30 min at 37°C [24]. U87 cells (2 × 10^4^ in 250 μl), MSC cells (2 × 10^4^ in 250 μl), and the cell mix (1 × 10^4^ of both cell types mixed in 250 μl) were incubated with EcTI (50 and 100 μM) for 15 min at 37°C in medium without FBS. Cell samples were seeded as monocultures and coculture into the coated insert, whereas 400 μl of medium with 10% FBS was added to the lower chamber. After 24 h at 37°C and 5% CO_2_, membranes were washed with PBS and cells were fixed for 30 min in cold methanol. Matrigel with the remaining cells was removed with a cotton swab from the upper surface of the chamber membrane. The invaded cells residing in the membrane's bottom surface were stained with 1% (w/v) toluidine blue for 30 min and washed with PBS. Cell invasion was quantified in an inverted microscope (Leica, Germany) by counting cells on at least 10 visual fields; imaging was recorded in a photography camera (3000 G – software Leica Application Suite EZ).

### Cell cycle and cell death assays

These assays were performed using 5 × 10^5^ U87 cells, 2 × 10^5^ MSC, or mixed cells samples (direct coculture condition) seeded in 6-well plates in DMEM containing 10% (v/v) FBS and incubated for 24 h at 37°C and 5% (v/v) CO_2_ [25]. Prior cell treatment, for cell cycle analysis, the cells were maintained in starvation with 0.2% (v/v) FBS for 24 h and treated with EcTI (50 and 100 μM) in DMEM with 10% FBS for an additional 24 h. Cells were trypsinized and centrifuged at 300 x g for 5 min and suspended in 300 μl of binding buffer containing 0.4 μl of Annexin APC (staining for early apoptotic cells) or 8 nM of Sytox Green (staining for dead cells and cell cycle) for each 60,000 cells. The flow cytometer analysis was performed in the FACS Aria III (BD, California, USA) using the Flow Jo 9 program.

### Calcium release measurements

These measurements were performed as described in Charles [26]. U87 cells (5 × 10^3^ cells/well) and MSC cells (2 × 10^3^ cells/well) were seeded into 96-well plates as monocultures and coculture (mixture with the same number of cells) in DMEM containing 10% (v/v) FBS for 24 h at 37°C and 5% (v/v) CO_2_. The medium was changed after this incubation and EcTI (50 and 100 μM) was added; plates were incubated for 24 h at the same conditions. Cells were subsequently washed with *Hank's Balanced Salt Solution* at pH 7.3 and incubated with Fura 2-AM (4 μM) and 0.02% (v/v) pluronic acid for 2 h. Fluorescence readings were performed with a FlexStation 3 Benchtop Multi-Model Microplate Reader, using λ_exc_ = 340/380 nm and λ_em_ = 505 nm.

### Determination of nitrite to nitric oxide (NO) conversion

This conversion rate was measured according to Dweik et al. [27]. U87 cells (5 × 10^3^ cells/well) and MSC (2 × 10^3^ cells/well) were seeded into 96-well plates as monocultures and coculture in DMEM containing 10% (v/v) FBS and incubated for 24 h at 37°C and 5% (v/v) CO_2_. The medium was changed after this incubation and EcTI (50 and 100 μM) was added; plates were incubated for 24 h at 37°C and 5% (v/v) CO_2_ and 25 μl of media was collected to determine the conversion of nitrite to nitric oxide by a chemoluminescence analysis performed with the Nitric Oxide Analyzer (NOA^TM^ 208i – Sievers). The value of FBS 10% NO release was excluded from the final NO concentration.

### Cytokine profiles of EcTI treated cells

U87 cells (5 × 10^5^) and MSC cells (2 × 10^5^) were seeded into 6-well plates as monocultures and coculture and allowed to attached for 24 h before fresh media with EcTI (50 and 100 μM) was added. After an additional 24 h of incubation, medium samples were collected for the determination of protein concentration using the MicroBCA assay with a standard bovine serum albumin (BSA from Thermo Scientific, Rockford, Illinois, USA). Protein samples were centrifuged at 1300 x g for 5 min at 4°C, and cytokines profiles were analyzed with the LuminexMAP [28]. For this, 40 μg of total proteins from each sample were loaded to the Milliplex MAP Human Cytokine/Chemokine Magnetic Bead Panel (HCYTMAG-60K-PX38), Merck Millipore, MA, USA. The sensitivity of this assay allows the detection of cytokines present in the concentration of pg/ml; FBS 10% was used as the blank.

### Western blotting

U87 cells (5 × 10^5^) and MSC (2 × 10^5^) were seeded into 6-well plates as monocultures and coculture (same number of mixed cells) in DMEM containing 10% (v/v) FBS and incubated for 24 h at 37°C and 5% (v/v) CO_2_. The medium was changed after this incubation and EcTI (50 and 100 μM) was added; plates were incubated for 24 h at 37°C and 5% (v/v) CO_2_. Cells were PBS washed, scraped in 500 μl of PBS containing 250 mM sodium orthovanadate, and centrifuged at 460 x g for 20 min at 4°C. Pelleted cells were lysed through vortexing and freeze/thaw cycles in 30 μl of lysis buffer [0.05 M Tris/HCl at pH 7.4, containing 0.25% (w/v) sodium deoxycholate, 1% (v/v) Tween 20, 10^-3^ M EGTA, 0.15 M NaCl, 10^-3^ M NaF, 10^-3^ M sodium orthovanadate, phosphatase (1:100), and protease inhibitors (1:100)] followed by incubation on ice for 2 h. Lysates were centrifuged at 25000 x g for 10 min at 4°C and the protein concentration was determined by the MicroBCA assay. Total protein in the cell lysate (30 μg) was separated by SDS-PAGE using 5% stacking and 15% separating gels. Separated proteins were transferred to PVDF membranes in buffer (0.05 M Tris, 0.384 M glycine, 30% (v/v) methanol) via 2 h and 40 min electrotransfer (0.2 ampers, BIO-RAD PowerPac HC, CA, USA). These membranes were subjected to blocking buffer [5% (w/v) BSA in 0.025 M Tris, 0.192 M glycine, and 0.1% (v/v) Tween 20] for 2 h. The membranes were subsequently washed with blocking buffer without BSA and incubated with primary antibodies against BR2, Src, p-Src^Y416^, Akt, p-Akt^S473^, β1 integrin and β-actin (1:1000), and FAK and p-FAK^Y397^ (1:500) diluted in blocking buffer for 16 h at 4°C. Membranes were washed with blocking buffer and incubated with anti-rabbit HRP-conjugated secondary antibody at a 1:1000 dilution (blocking buffer) for 2 h at room temperature. After washing membranes with 0.025 M Tris and 0.192 M glycine, the Super Signal^®^ West Pico Chemo-luminescent substrate was added and incubated for 5 min. The signal was detected using the chemo-luminescence Bio-Imaging Systems in the GelCapture program; the densitometry analysis was performed using the Image J with the ratio phosphorylated form vs total protein and β-actin normalization in each sample.

### Quantitative real-time PCR analyses

U87 cells (5 × 10^4^) were seeded into 24-well plates and incubated for 24 h; medium was replaced with fresh medium (control) containing EcTI (2.5 μM, 5 μM, 10 μM, 20 μM, and 100 μM) and plates were incubated for 72 h at 37°C and 5% (v/v) CO_2_. Total RNA was isolated from three biological replicates of every treatment condition using the Trizol reagent (Invitrogen Ltd., Paisley, UK) according to the manufacturer's instructions. RNA was purified using the RNeasy Mini kit (Qiagen, UK) and RNA integrity was confirmed using the Agilent 2100 Bioanalyzer (Agilent Technologies, USA). cDNA was generated using 1 μg of total RNA and the High-Capacity cDNA Reverse Transcription kits (Applied Biosystems, USA) in 50 μl volume reactions according to the manufacturer's protocol. The expression of cell cycle genes (*Ccnd1*, *Cdkn1a*, *Tp53*, *Bax*, and *Bcl-2*), metalloproteases (*Mmp14*, *Mmp2*, and *Mmp9*), serine proteases (*Plaur* and *Plau*), calpains (*Capn1* and *Capn2*), and cathepsins (*CtsB* and *CtsL*) were quantified using real-time quantitative PCR (ABI 7900 HT Sequence Detection System, Applied Biosystems, USA). The real-time PCR reactions were performed using 1:10 dilutions (1 μl/well) of each cDNA sample, TaqMan Universal PCR Master Mix (Applied Biosystems, USA), and TaqMan Gene Expression assays (all from Applied Biosystems, USA) for: *Ccnd1* (Cyclin D1), Hs00765553_m1; *Cdkn1A* (cyclin-dependent kinase inhibitor 1A (p21, Cip1)), Hs00355782_m1; *Tp53* (tumor protein p53), Hs01034249_m1; *Bax* (Bcl-2 associated X protein), Hs99999001_m1; Bcl2 (B-cell CLL/lymphoma2), Hs00608023_m1; *Mmp14* (matrix metallopeptidase 14 (membrane-inserted)), Hs00237119_m1; *Mmp2* (matrix metallopeptidase 2), Hs1548727_m1; *Mmp9* (matrix metallopeptidase 9), Hs00234579_m1; *Plaur* (plasminogen activator, urokinase receptor), Hs00958880_m1; *Plau* (plasminogen activator, urokinase), Hs01547054_m1; *Capn1* (calpain 1, (mu/l) large subunit), Hs00559804_m1; *Capn2* (calpain 2, (m/II) large subunit), Hs00965097_m1; *CtsB* (cathepsin B), Hs00947433; and *CtsL* (cathepsin L), Hs00964650_m1. The amplification of the *Gadph* (glyceraldehyde 3-phosphate dehydrogenase) probe, a pre-developed TaqMan assay no. 4310884E, was performed as the internal control. The SDS v2.2 software (Applied Biosystems) was used to analyze the data according to the comparative Ct method [29].

### Statistical analyses

All experiments were performed in triplicates and independently repeated at least three times unless otherwise stated. The statistical analyses of the data were performed by one-way ANOVA followed by the Tukey's test. The *p-value* < 0.05 was considered significant. Data were expressed as means ± standard deviation (SD).

## Abbreviations

BAPA: Bz-Arg-pNa substrate
Bax: Bcl-2 associated X protein
Bcl2: B-cell CLL/lymphoma2
BR2: bradykinin receptor 2
BrDU: bromodeoxyuridine
Ca^2+^: calcium
Capn1: calpain 1
Capn2: calpain 2
CCL2/MCP-1: monocyte chemoattractant protein-1
Ccnd1: cyclin D1
Cdkn1A: cyclin-dependent kinase inhibitor 1A (p21, Cip1)
CtsB: cathepsin B
CtsL: cathepsin L
ECM: extracellular matrix
EcTI: *Enterolobium contortisiliquum* Trypsin Inhibitor
G418: geneticin
Gadph: glyceraldehyde 3-phosphate dehydrogenase
GBM: glioblastoma multiforme
G-CSF: granulocyte colony-stimulating factor
GM-CSF: granulocyte macrophage colony-stimulating factor
MSC: mesenchymal stem cells
MTT: 3-(4,5-dimethylthiazol-2-yl)-2,5-diphenyltetrazolium bromide
NO: oxide nitric
Plau: plasminogen activator urokinase
Plaur: plasminogen activator urokinase receptor
TFA: trifluoroacetic acid
Tp53: tumor protein p53
U87/MSC: direct coculture

## References

[1] Seymour T, Nowak A, Kakulas F (2015). Targeting Aggressive Cancer Stem Cells in Glioblastoma. Front Oncol.

[2] Aboody KS, Najbauer J, Metz MZ, D’Apuzzo M, Gutova M, Annala AJ, Synold TW, Couture LA, Blanchard S, Moats RA, Garcia E, Aramburo S, Valenzuela VV (2013). Neural stem cell-mediated enzyme/prodrug therapy for glioma: preclinical studies. Sci Transl Med.

[3] Altaner C, Altanerova V, Cihova M, Ondicova K, Rychly B, Baciak L, Mravec B (2014). Complete regression of glioblastoma by mesenchymal stem cells mediated prodrug gene therapy simulating clinical therapeutic scenario. Int J Cancer.

[4] Barcellos-de-Souza P, Gori V, Bambi F, Chiarugi P (2013). Tumor microenvironment: bone marrow-mesenchymal stem cells as key players. Biochim Biophys Acta.

[5] Tajnšek U, Motaln H, Levičar N, Rotter A, Lah TT (2013). The Duality of Stem Cells: Double-Edged Sword in Tumor Evolution and Treatment. Trends in Stem Cell Proliferation and Cancer Research.

[6] Motaln H, Turnsek TL (2015). Cytokines play a key role in communication between mesenchymal stem cells and brain cancer cells. Protein Pept Lett.

[7] Chanda D, Isayeva T, Kumar S, Hensel JA, Sawant A, Ramaswamy G, Siegal GP, Beatty MS, Ponnazhagan S (2009). Therapeutic potential of adult bone marrow-derived mesenchymal stem cells in prostate cancer bone metastasis. Clin Cancer Res.

[8] Motaln H, Schichor C, Lah TT (2010). Human mesenchymal stem cells and their use in cell-based therapies. Cancer.

[9] Mercapide J, Rappa G, Lorico A (2012). The intrinsic fusogenicity of glioma cells as a factor of transformation and progression in the tumor microenvironment. Int J Cancer.

[10] Schichor C, Albrecht V, Korte B, Buchner A, Riesenberg R, Mysliwietz J, Paron I, Motaln H, Turnšek TL, Jürchott K, Selbig J, Tonn JC (2012). Mesenchymal stem cells and glioma cells form a structural as well as a functional syncytium *in vitro*. Exp Neurol.

[11] Olson OC, Joyce JA (2015). Cysteine cathepsin proteases: regulators of cancer progression and therapeutic response. Nat Rev Cancer.

[12] Luo R, Wang X, Dong Y, Wang L, Tian C (2014). Activation of protease-activated receptor 2 reduces glioblastoma cell apoptosis. J Biomed Sci.

[13] Sevenich L, Joyce JA (2014). Pericellular proteolysis in cancer. Genes Dev.

[14] Turk B (2006). Targeting proteases: successes, failures and future prospects. Nat Rev Drug Discov.

[15] Ferreira RS, Zhou D, Ferreira JG, Silva MC, Silva-Lucca RA, Mentele R, Paredes-Gamero EJ, Bertolin TC, Dos Santos Correia MT, Paiva PM, Gustchina A, Wlodawer A, Oliva ML (2013). Crystal Structure of Bark Protein (CrataBL) and Its Effect in Human Prostate Cancer Cell Lines. PLoS One.

[16] Oliva ML, Sampaio MU (2009). Action of plant proteinase inhibitors on enzymes of physiopathological importance. An Acad Bras Cienc.

[17] Silva MC, de Paula CA, Ferreira JG, Paredes-Gamero EJ, Vaz AM, Sampaio MU, Correia MT, Oliva ML (2014). Bauhinia forficata lectin (BfL) induces cell death and inhibits integrin-mediated adhesion on MCF7 human breast cancer cells. Biochim Biophys Acta.

[18] de Paula CA, Coulson-Thomas VJ, Ferreira JG, Maza PK, Suzuki E, Nakahata AM, Nader HB, Sampaio MU, Oliva ML (2012). Enterolobium contortisiliquum trypsin inhibitor (EcTI), a plant proteinase inhibitor, decreases *in vitro* cell adhesion and invasion by inhibition of Src protein-focal adhesion kinase (FAK) signalling pathways. J Biol Chem.

[19] Nakahata AM, Mayer B, Ries C, de Paula CA, Karow M, Neth P, Sampaio MU, Jochum M, Oliva ML (2011). The effects of a plant proteinase inhibitor from Enterolobium contortisiliquum on human tumor cell lines. Biol Chem.

[20] Gravett PS, Viljoen CC, Oosthuizen MM (1991). A steady-state kinetic analysis of the reaction between arginine esterase E-I from Bitis gabonica venom and synthetic arginine substrates and the influence of pH, temperature and solvent deuterium isotope. Int J Biochem.

[21] Pillat MM, Oliveira MN, Motaln H, Breznik B, Glaser T, Lah TT, Ulrich H (2016). Glioblastoma-mesenchymal stem cell communication modulates expression patterns of kinin receptors: Possible involvement of bradykinin in information flow. Cytometry A.

[22] Mosmann T (1983). Rapid colorimetric assay for cellular growth and survival: application to proliferation and cytotoxicity assays. J Immunol Methods.

[23] Okegawa T, Pong RC, Li Y, Hsieh JT (2004). The role of cell adhesion molecule in cancer progression and its application in cancer therapy. Acta Biochim Pol.

[24] Chen HC (2005). Boyden chamber assay. Methods Mol Biol.

[25] Vermes I, Haanen C, Steffens-Nakken H, Reutelingsperger C (1995). A novel assay for apoptosis. Flow cytometric detection of phosphatidylserine expression on early apoptotic cells using fluorescein labelled Annexin V. J Immunol Methods.

[26] Charles A (1998). Intercellular calcium waves in glia. Glia.

[27] Dweik RA, Laskowski D, Abu-Soud HM, Kaneko F, Hutte R, Stuehr DJ, Erzurum SC (1998). Nitric oxide synthesis in the lung. Regulation by oxygen through a kinetic mechanism. J Clin Invest.

[28] Kucera R, Topolcan O, Treskova I, Kinkorova J, Windrichova J, Fuchsova R, Svobodova S, Treska V, Babuska V, Novak J, Smejkal J (2015). Evaluation of IL-2, IL-6, IL-8 and IL-10 in Malignant Melanoma Diagnostics. Anticancer Res.

[29] Breznik B, Motaln H, Vittori M, Rotter A, Lah Turnšek T (2017). Mesenchymal stem cells differentially affect the invasion of distinct glioblastoma cell lines. Oncotarget.

[30] Wolf K, Friedl P (2011). Extracellular matrix determinants of proteolytic and non-proteolytic cell migration. Trends Cell Biol.

[31] Abbas T, Dutta A (2009). p21 in cancer: intricate networks and multiple activities. Nat Rev Cancer.

[32] Alao JP (2007). The regulation of cyclin D1 degradation: roles in cancer development and the potential for therapeutic invention. Mol Cancer.

[33] Shimura T, Hamada N, Sasatani M, Kamiya K, Kunugita N (2014). Nuclear accumulation of cyclin D1 following long-term fractionated exposures to low-dose ionizing radiation in normal human diploid cells. Cell Cycle.

[34] Ljubimova JY, Fujita M, Khazenzon NM, Ljubimov AV, Black KL (2006). Changes in laminin isoforms associated with brain tumor invasion and angiogenesis. Front Biosci.

[35] Lu X, Kang Y (2009). Cell fusion as a hidden force in tumor progression. Cancer Res.

[36] Mentlein R, Hattermann K, Held-Feindt J (2012). Lost in disruption: role of proteases in glioma invasion and progression. Biochim Biophys Acta.

[37] Mrugala MM (2013). Advances and challenges in the treatment of glioblastoma: a clinician’s perspective. Discov Med.

[38] Bouchard V, Harnois C, Demers MJ, Thibodeau S, Laquerre V, Gauthier R, Vézina A, Noël D, Fujita N, Tsuruo T, Arguin M, Vachon PH (2008). B1 integrin/Fak/Src signalling in intestinal epithelial crypt cell survival: integration of complex regulatory mechanisms. Apoptosis.

[39] Arias-Salgado EG, Lizano S, Sarkar S, Brugge JS, Ginsberg MH, Shattil SJ (2003). Src kinase activation by direct interaction with the integrin beta cytoplasmic domain. Proc Natl Acad Sci U S A.

[40] Thiyagarajan V, Tsai MJ, Weng CF (2015). Antroquinonol Targets FAK-Signalling Pathway Suppressed Cell Migration, Invasion, and Tumor Growth of C6 Glioma. PLoS One.

[41] Brindle NR, Joyce JA, Rostker F, Lawlor ER, Swigart-Brown L, Evan G, Hanahan D, Shchors K (2015). Deficiency for the cysteine protease cathepsin L impairs Myc-induced tumorigenesis in a mouse model of pancreatic neuroendocrine cancer. PLoS One.

[42] Montana V, Sontheimer H (2011). Bradykinin promotes the chemotactic invasion of primary brain tumors. J Neurosci.

[43] Seifert S, Sontheimer H (2014). Bradykinin enhances invasion of malignant glioma into the brain parenchyma by inducing cells to undergo amoeboid migration. J Physiol.

[44] Leloup L, Mazeres G, Daury L, Cottin P, Brustis JJ (2006). Involvement of calpains in growth factor-mediated migration. Int J Biochem Cell Biol.

[45] Guevara-Lora I, Blonska B, Faussner A, Kozik A (2013). Kinin-generating cellular model obtained from human glioblastoma cell line U-373. Acta Biochim Pol.

[46] Wang YB, Peng C, Liu YH (2007). Low dose of bradykinin selectively increases intracellular calcium in glioma cells. J Neurol Sci.

[47] Safdar S, Payne CA, Tu NH, Taite LJ (2013). Targeted nitric oxide delivery preferentially induces glioma cell chemosensitivity via altered p53 and O(6)-methylguanine-DNA methyltransferase activity. Biotechnol Bioeng.

[48] Prevarskaya N, Skryma R, Shuba Y (2011). Calcium in Tumor metastasis: new roles for known actors. Nat Rev Cancer.

[49] Swaroop GR, Kelly PA, Bell HS, Shinoda J, Yamaguchi S, Whittle IR (2000). The effects of chronic nitric oxide synthase suppression on glioma pathophysiology. Br J Neurosurg.

[50] Deb TB, Coticchia CM, Dickson RB (2004). Calmodulin-mediated activation of Akt regulates survival of c-Myc-overexpressing mouse mammary carcinoma cells. J Biol Chem.

[51] Dimmeler S, Fleming I, Fisslthaler B, Hermann C, Busse R, Zeiher AM (1999). Activation of nitric oxide synthase in endothelial cells by Akt-dependent phosphorylation. Nature.

[52] Xu N, Lao Y, Zhang Y, Gillespie DA (2012). Akt: a double-edged sword in cell proliferation and genome stability. J Oncol.

[53] Piperi C, Zisakis A, Lea RW, Kalofouti A (2005). Role of Cytokines in the Regulation of Glioma Tumour Growth and Angiogenesis. Am J Immunol.

[54] Zhu VF, Yang J, Lebrun DG, Li M (2012). Understanding the role of cytokines in Glioblastoma Multiforme pathogenesis. Cancer Lett.

[55] Motaln H, Gruden K, Hren M, Schichor C, Primon M, Rotter A, Lah TT (2012). Human mesenchymal stem cells exploit the immune response mediating chemokines to impact the phenotype of glioblastoma. Cell Transplant.

[56] Oliveira MN, Pillat MM, Motaln H, Ulrich H, Lah TT, Kos J, Poklar-Ulrich N (2015). Kinin receptor expression and activity in co-cultures of mesenchymal stem and glioblastoma cells. Molecules of life: Book of abstracts, FEBS3+ Meeting.

